# The Majority of Resorptions in Old Mice Are Euploid

**DOI:** 10.1371/journal.pone.0143360

**Published:** 2015-12-04

**Authors:** Yong Tao, X. Johné Liu

**Affiliations:** 1 Ottawa Hospital Research Institute, The Ottawa Hospital—General Campus, 501 Smyth Road, Box 511, Ottawa, Ontario, K1H 8L6, Canada; 2 Department of Obstetrics and Gynaecology, and Department of Biochemistry, Microbiology and Immunology, University of Ottawa, Ottawa, Ontario, Canada; University of Science and Technology of China, CHINA

## Abstract

Chromosomal abnormality is a leading cause of aging-related infertility, spontaneous abortion and congenital birth defects in humans. Karyotype analyses of spontaneously aborted human fetuses reveal high proportions (~50%) being chromosomal abnormal with the majority being trisomies of various chromosomes. As a model organism, mice are widely used for studies of reproduction and reproductive aging. Like older women, older mice exhibit high incidences of early embryo death. However, it is not known if aneuploidy is prevalent amongst resorptions in older mice. We have karyotyped 65 retarded/resorbed fetuses in 10-month-old C57BL/6 mice, and found that 55 (84.6%±8.8%, with 95% confidence) were euploid. Similarly, of 40 such fetuses from 17 month-old C57BL/6 mice, we found 38 (95±7%, with 95% confidence 95%) being euploid. Therefore, aneuploidy is not a leading cause of embryo death in older mice.

## Introduction

While less than 10% of natural pregnancies result in spontaneous abortion for women in their early 20s, more than 50% end in spontaneous abortions for women age 40 and older [1]. Very similar overall spontaneous abortion rates and maternal age association are found in ART (assisted reproductive technology) patients using autologous eggs [2,3]. On the other hand, spontaneous abortion risk among pregnancies conceived with donor eggs is low (13%) with little variation by patient age, indicating that egg quality is the determining factor in miscarriage risk [2].

Aneuploidy is thought to be a leading cause of spontaneous abortions in humans, present in 50% (30%-64%) of analyzed abortuses, with the majority (~2/3) being trisomies of various chromosomes [4–7]. In addition to miscarriage, significant proportions of human conceptions are lost prior to the recognition of pregnancy [8,9], and egg aneuploidy is thought to be the leading cause of these “pre-clinical abortion” [10].

This woman-specific reproductive aging likely arises due to the peculiar oogenesis (the process of generating eggs) in all mammalian species, plus our longevity. Unlike males that continuously generate mature sperms from germ line stem cells throughout adulthood, oogenesis begins during the embryonic stage when germ line stem cells initiate meiosis and develop into primary oocytes. By birth, the females have developed a finite number of primary oocytes that comprises their lifetime egg supply. These oocytes are arrested in meiotic prophase, containing chromosome bivalents each having four chromatids linked together by a combination of sister chromatid cohesion and non-sister cross-over, with this linkage persisting for decades in women. In meiosis I, the two sister chromatids are segregated to the same cell (mature egg or 1^st^ polar body) in a process called oocyte maturation; at fertilization, the sisters are segregated between the 2^nd^ polar body and the fertilized egg in meiosis II [11]. As females age, chromosome cohesion becomes progressively weakened [12], predisposing the oocytes to greater risk of premature separation of sister chromatids (PSSC) during meiosis I, hence producing aneuploid eggs [12–18].

As a model organism, mice are widely used for studies of reproduction and reproductive aging. In both humans and in mice, aneuploidy is rarely survivable in pregnancy. It is estimated that only 0.3% of live births in humans is aneuploid [10]. Similarly, Goodlin found no aneuploidy in analyzing 750 live births to 1.5-year-old F1 females from BALBc/129 crosses [19]. Like women, female mice experience significantly higher incidence of embryo death as they age [20]. However, it is not known how much of this is due to aneuploidy. In this study, we have undertaken karyotype analyses of resorbed and retarded fetuses on 9.5 dpc in 10 month-old and 17-month-old C57BL/6 mice. Our results indicated that majority of the resorbed and retarded fetuses in these mice were euploid.

## Materials and Methods

### Animals and animal procedures

Animal protocols were approved by the Animal Care Committee of the Ottawa Hospital Research Institute and University of Ottawa. C57BL/6 retired breeders (8–9 months of ages on delivery, each batch identified by the month of birth) were purchased from Taconic (Hudson, NY). Young (8 weeks of age) fertile CF1 males were from Charles River (St-Constant, QC, Canada).

Females were supplied with male bedding (Whitten effect) and with either 1% putrescine in drinking water or control drinking water for 2 days prior to mating to fertile males. Putrescine and the male were withdrawn when a vaginal plug was seen in the morning, or following two consecutive nights of mating. Plugged mice were sacrificed, by cervical dislocation, on 9.5 dpc.

### Karyotyping mouse resorption sites and embryos

**Fig 1:**
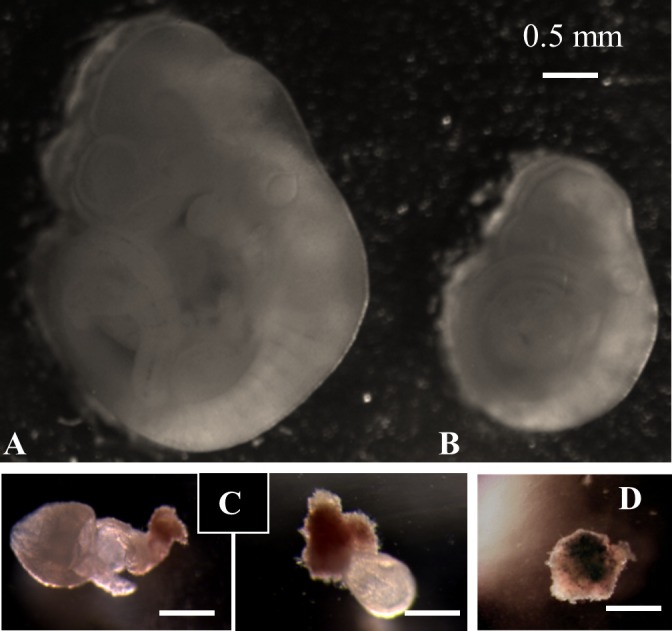
Fetal classification on 9.5 dpc in older C57BL/6 mice. C57BL/6 mice (10–17 months of age) were killed on 9.5 dpc. All implants were dissected for determining fetal states. A. A normal 9.5 dpc fetus. B. A retarded littermate. C. Two examples of Grade 1 resorption. D. A Grade 2 resorption. Scale bar = 0.5 mm.

Individual implantation sites were dissected according to Pereira et al [21] and then embryos/fetal tissues were recovered for embryo grading and karyotype analyses. The embryo/resorptions were classified as follows (Fig 1).

Normal fetuses: Fetuses with typical morphology at the Theiler stage 15 [22].

Retarded fetuses: These fetuses appeared intact but were considerably smaller than their normal littermates.

Resorptions: Implantation sites containing no intact fetus inside the placenta. They were further ranked as follows.

Grade 1 resorption: Resorption sites that contained grossly abnormal/partially resorbed fetal tissues (including fetal membranes).

Grade 2 resorption: Resorption sites with no fetal membranes but only residual ectoplacental cone.

Implants were dissected in Petri dishes containing of αMEM plus 1 μM nocodazole. To karyotype a normal or developmentally retarded fetus, placenta and surrounding tissues were carefully removed, and the fetal membranes (visceral yolk sac and amniotic membranes) [21] were freed from the fetus and used for karyotyping. To karyotype Grade 1 resorptions, both fetal membranes and partially resorbed fetal tissues, if any, were used. For Grade 2 resorptions, only the residual ectoplacental cone was used. Membranes and other fetal tissues were incubated in αMEM plus 1 μM nocodazole in 24-well plate at 37C 5% CO_2_ for 2h before being subjected to chromosome spreading according to Evans et al. [23]. The slides were stained with 0.1% SYTOX green (Invitrogen, Eugene, Oregon) before being imaged by epifluorescence microscopy. Chromosomes were counted to identify the karyotypes of the fetuses/resorptions. To avoid bias, the slides were coded before being examined.

### Statistics

Embryo mosaicism data were analyzed by Fisher’s exact test, two-tailed.

## Results

Older C57BL/6 mice (8–11 months of age) exhibit modest egg aneuploidy rates, 2.6%-12.7% [18,24]. Ten (10) month-old C57BL/6 mice however exhibit extraordinarily high level of early embryo death (60%) [25], implying that aneuploidy might not be the leading cause of embryo death in older mice.

To address this directly, we recovered all severely retarded (Fig 1B) and resorbed (Fig 1C and 1D) implants at 9.5 dpc from the 10-month-old C57BL/6 mice [25], and subjected them for karyotype analyses. To facilitate karyotype analyses, we further classified resorptions into two grades, with Grade 1 having some fetal tissues (Fig 1C) and Grade 2 with only remnants of ectoplacental cone (Fig 1D). Ectoplacental cone remnants yielded few or no metaphase spreads in 10-month-old mice (S1 Table) or 17-month-old mice (S2 Table), suggesting that the ectoplacental cone remnants contained few or no mitotic cells at the time of dissection (9.5 dpc). Therefore Grade 2 resorptions did not produce any positive karyotype.

**Fig 2:**
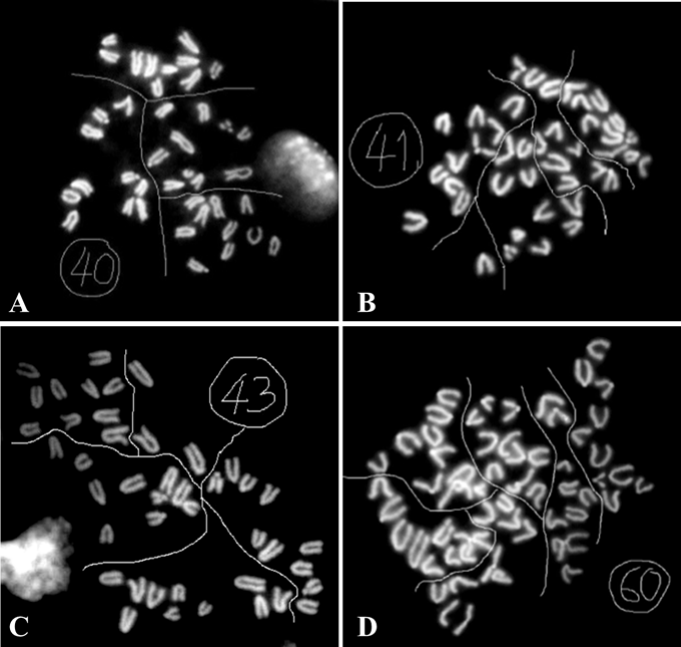
Typical images of metaphase chromosome spreads. A. A euploid cell with 40 chromosomes. B. A hyperploid cell with 41chromosomes. C. A hyperploid cell with 43 chromosomes. D. A triploid cell with 60 chromosomes. Lines are drawn to divide the spreads into 10-chromosome blocks, with the exception of the extra chromosomes in hyperploid karyotypes, to facilitate chromosome counting.

The minimum countable metaphase spreads for a positive karyotype were set at 12; a specific karyotype (euploid, hyperploids or triploid, see Fig 2) was assigned based on the predominant karyotype amongst the countable metaphase spreads (Table 1 and Table 2; Details can be found in S1 Table and S2 Table). A total of 54 retarded fetuses/resorptions were recovered from 10-month-old C57BL/6 mice (Table 1, “CTRL”), yielding 37 positive karyotypes (all but one in the two groups: retarded and Grade 1 resorption).

**Table 1:** Karyotypes of retarded/resorbed fetuses in 10-month-old C57BL/6 mice.

Group	Total	Abnormal implants			Normal implants karyotyped (eu, hyper, tri)
		Retarded (eu, hyper, tri)	Grade1 resorption (eu, hyper, tri)	Grade 2 resorption (% abnormal)	
CTRL	54	17* (13, 2, 1)	21 (21, 0, 0)	16 (29.6)	8 (8, 0, 0)
PUT	37	17 (14, 2, 1)	11 (7, 1, 3)	9 (24.3)	13 (13, 0, 0)
Total	91	34 (27, 4, 2)	32 (28, 1, 3)	25 (27.5)	21 (21, 0, 0)

Numbers within parentheses indicate identified karyotypes: eu = euploid; hyper = hyperploids; tri = triploid.

*: One retarded fetus generated only 8 countable spreads, not reaching the threshold of 12 countable spreads to be included in our analyses, although this fetus was likely euploid (mosaic) (1 × 41, 6 × 40, 1× 38). Details can be found in S1 Table.

**Table 2:** Karyotypes of retarded/resorbed fetuses in 17-month-old C57BL/6 mice.

Mice	Pregnancies	Implants/ pregnancies	Abnormal implants			Karyotyped (40/87)		
			Retarded	Grade1	Grade 2	euploid	hyperploid	triploid
42	14 (33.3%)	87/14 = 6.2	6	46	35 (40%)	38 (95.0%)	1 (2.5%)	1 (2.5%)

No normal fetuses were found in these mice. Details can be found in S2 Table.

The majority, 34 (92±9%, with 95% confidence), were euploid. The rest were hyperploids (2 or 5.4%) and triploid (1 or 2.7%). Therefore, notwithstanding the 16 Grade 2 resorptions which yielded few or no metaphase spreads, our results indicated that the majority of retarded/resorptions in 10-month-old C57BL/6 mice were euploid.

Peri-ovulatory putrescine supplementation significantly reduces embryo resorptions in 10-month-old C57BL/6 mice [25]. We also recovered all severely retarded and resorbed implants (total 37; Table 1, “PUT”) in the putrescine group for karyotype analyses. All 28 retarded implants and Grade 1 resorptions produced positive karyotypes, 21 of which (75±16%, with 95% confidence) were euploid with the rest being hyperploid (3 or 10.7%) and triploid (4 or 14.3%). There was no statistical difference between the proportion of euploid karyotypes (21/28 or 75%) in the putrescine group and that (34/37 or 92%) in the control group (P = 0.9 by Fisher’s exact test). These numbers, while too small for determining the possible effect of peri-ovulatory putrescine supplementation on aneuploid conceptions, nonetheless indicated that the majority of retarded/resorbed fetuses in putrescine-treated mice, like in control mice, were euploid.

Combining both groups, we karyotyped a total of 65 retarded/resorbed fetuses in 10-month-old C57BL/6 mice and found 55 (84.6%±8.8) were euploid, with the rest equally splitting between hyperploid (7.7%) and triploid (7.7%) (Table 1, “Total”). We also karyotyped 21 normal fetuses (Fig 1A) and found that all 21 were euploid (Table 1, far left column).

To further investigate the prevalence of aneuploidies among resorptions in older mice, we subjected 17-month-old C57BL/6 mice to similar mating and karyotype analyses, except that none were treated with putrescine supplementation. All 42 females were killed on 9.5 dpc, yielding 14 pregnancies with average 6.2 implants per pregnancy, compared to 7.6 implants per pregnancy in 10-month-old mice [25]. All implants, 87 in total, were identified as severely retarded or resorbed fetuses, meaning that no live birth would be anticipated if pregnancy were allowed to term [25]. We successfully karyotyped 40 of these implants and found 38 (95±7%, with 95% confidence) euploids, with the rest being hyperploid (1 or 2.5%) or triploid (1 or 2.5%) (Table 2; Details can be found in S2 Table). Therefore, similar to 10-month-old mice, the majority of retarded/resorbed fetuses in 17-month-old mice were euploid.

We found no hypoploid fetuses, in which the dominant karyotype contains 39 or fewer chromosomes, in this study, suggesting that hypoploid fetuses, if present, may be amongst the Grade 2 resorptions.

All together, we have successfully karyotyped a total of 105 retarded/resorbed fetuses in 10-17-month-old C57BL/6 mice. The vast majority of them (93, or 88.6%) were euploid. Interestingly, we found that half (46/93 or 49.5%) of these euploid retarded/resorbed fetuses had at least one (range: 1–4) hyperploid cells. The presence of hyperploid cells in euploid embryos indicated mosaicism, the product of mitotic errors during embryogenesis. (This is in contrast to the presence of hypoploid spreads which could be due to mosaicism or, more likely, chromosome loss during the karyotyping procedure [24].) In contrast, only four such mosaic euploids were found amongst the 21 normal fetuses (4/21 or 19%; *P* = 0.0143 by Fisher’s exact test) (Table 3; Details can be found in S1 Table and S2 Table). Similar numbers of countable spreads were analyzed in the normal fetuses (34±12) and the retarded/resorbed fetuses (36±12), suggesting that the higher degree of mosaicism identified in the retarded/resorbed fetuses was not an experimental artifact.

**Table 3:** Mosaicism amongst euploid normal and euploid abnormal embryos.

Group	Normal (34±12)		Abnormal (36±12)	
	Non-mosaic	Mosaic	Non-mosaic	Mosaic
10-mon CTRL	6	2	18	16
10-mon PUT	11	2	10	11
17-mon CTRL	/	/	19	19
Total	17 (81.0%)	4 (19.0%)	47 (50.5%)	46 (49.5%)
**Fisher’s exact test**	***P* = 0.0143**			

The numbers on top row indicate average numbers of countable spreads with standard deviation.

## Discussion

In the karyotyping protocol [23], fetal membranes and partially resorbed fetal tissues were treated with nocodazole for two hours followed by immediate tissue/cell disruption and chromosome spreading. Given the limited fetal tissues, many resorptions did not produce more than a few dozen metaphase spreads, and some even fewer. Although we included any fetuses/resorptions with 12 or more countable spreads in our karyotype analyses, most (>90%) had 20 or more countable spreads, giving a high degree of certainty in our karyotype assignment.

With 100% of Grade 1 resorptions and all but one retarded fetuses positively karyotyped in the 10-month-old mice, it is clear that the majority (84.6%) were euploid. We found 5 (7.7%) each of hyperploid and triploid, but no hypoploid fetuses. The absence of hypoploid (monosomy) amongst the karyotyped implants suggested that monosomic mouse embryos may be amongst Grade 2 resorptions which had only ectoplacental cone remnants left on 9.5 dpc and which produced no reliable karyotypes. This is consistent with earlier studies indicating that monosomic mouse embryos die at or shortly after implantation [26–28] whereas trisomic (hyperploid) embryos can survive much longer [28,29]. However, it is very unlikely that the majority of Grade 2 resorptions were hypoploid since hypoploid conceptions cannot be more prevalent than hyperploid conceptions. Regardless, the lack of karyotype information on Grade 2 resorptions would not jeopardize the conclusion that the majority of retarded/resorbed fetuses were euploid because Grade 2 resorptions constituted a minority of the abnormal fetuses (25/91 or 27.5%) in 10-month-old mice.

Despite the greater resorption rate in the 17-month-old C67BL/6 mice (in fact 100% the implants were either severely retarded or resorbed, compared to 60% for 10-month-old [25]), we did not find higher aneuploidy rates amongst the implants in these mice. One caveat is the greater proportion of the resorptions (54.0%) in the 17-month-old mice that did not yield a positive karyotype, mostly due to the larger proportion being Grade 2 resorptions. Although we cannot rule out the possibility of higher aneuploidy prevalence amongst these resorptions, aneuploidy is not likely the major cause for their early demise. In fact, we found as many triploids (trisomic for all 20 chromosomes) as trisomies (typically trisomic for one chromosome) amongst the abnormal fetuses in 10-17-month-old mice. The survivability of triploid embryos suggest that trisomy of a single chromosome will not likely cause early demise, as demonstrated for many chromosomes [28,29]. On the other hand, monosomies might cause early demise but their prevalence should not exceed that of trisomies.

Similar to our finding in mice, the occurrence of triploid spontaneous abortuses in humans is second only to trisomies in frequency amongst chromosome abnormalities [5,6]. However, unlike aneuplodies (trisomies and monosomies) which are chromosome segregation errors and which are mostly egg-derived, most triploidies appear to be due paternal factors, especially dispermy, in humans [30,31] and in mice [32].

Mosaicism is very common in early human embryos [33,34], and it is thought to be responsible for poor fertility performance in humans [35]. Our observation of a greater proportion of mosaicism amongst the retarded/resorbed fetuses, compared to normal fetuses, suggest that even the much lower levels of embryo mosaicism (typically one hyperploid cell amongst at least 20 countable spreads) found in 9.5 dpc mouse embryos may have a negative impact in development.

The relatively low proportion of aneuploidy amongst resorptions in older mice suggests relatively low incidences of aneuploid conceptions in these mice. Given that aneuploidy does not affect pre-implantation development in mice [36], our data are consistent with the modest egg aneuploidy rates found in older C57BL/6 mice [18,24]. However, in contrast to the general agreement that egg aneuploid rates in women 40 and older are in excess of 50% [14,37–39], the reported egg aneuploidy rates in older mice vary greatly, from 2–3% [24,40] to 60% [41] and anywhere in between [16,18,42–44]. Apart from age and mouse strain differences, this variation likely reflects the complexity of egg aneuploidies (whole chromosome nondisjunction and various forms of premature separation of sister chromatids [16,17,45]) and the technical limitations of currently applied cytogenetic methods (for egg karyotyping).

Our data highlight the importance of aneuploidy-independent factors contributing to the developmental potential of aged oocytes. Oocyte “cytoplasmic maturation” [46] likely plays critical roles in determining the developmental potential of aged oocytes in mice and in humans [45]. Understanding factors contributing to resorption in mice is thus critical for understanding and treating reproductive aging.

## Supporting Information

S1 Table Details of karyotypes of retarded/resorbed fetuses in 10-month-old C57BL/6 mice. (DOCX)

S2 Table Details of karyotypes of retarded/resorbed fetuses in 17-month-old C57BL/6 mice. (DOCX)
