# Supplementary material for: The Majority of Resorptions in Old Mice Are Euploid
Source: PLoS One. 2015 Dec 4;10(12):e0143360. doi: 10.1371/journal.pone.0143360 (PMC4670076; doi:10.1371/journal.pone.0143360)
Supplement: S1 Table — (DOCX) [file pone.0143360.s001.docx]

| **Fetal code** | **Fetal status** | **countable cells*** | **Karyotype in #dyads** | | | | **Diagnosis** |
| --- | --- | --- | --- | --- | --- | --- | --- |
|  |  |  | **60**** | **41***** | **40** | **≤39** |  |
| Control 1 | 1 | 29 | 0 | 0 | 25 | 4 | euploid |
| Control 2 | 2 | / | / | / | / | / | / |
| Control 3 | 1 | 32 | 0 | 1 | 24 | 7 | euploid (mosaic) |
| Control 4 | 1 | 33 | 0 | 1 | 28 | 4 | euploid (mosaic) |
| Control 5 | retarded | 28 | 19/28 | 0 | 0 | 0 | triploid |
| Control 6 | 1 | 24 | 0 | 1 | 12 | 11 | euploid (mosaic) |
|  |  |  | 41, 1×; 40, 12×; 39, 8×; ≤38, 3× | | | | |
| Control 7 | 1 | 31 | 0 | 0 | 24 | 7 | euploid |
| Putrescine 1 | normal | 29 | 0 | 0 | 21 | 8 | euploid |
| Putrescine 2 | 1 | 35 | 0 | 0 | 28 | 7 | euploid |
| Control 8 | 1 | 30 | 0 | 0 | 26 | 4 | euploid |
| Control 9 | 1 | 25 | 0 | 3/4 | 12 | 9 | euploid (mosaic) |
| Control 10 | retarded | 43 | 0 | 38 | 1 | 4 | hyperploid (41) |
| Control 11 | 1 | 38 | 0 | 1 | 32 | 5 | euploid (mosaic) |
| Control 12 | 1 | 33 | 0 | 0 | 20 | 13 | euploid |
| Control 13 | 2 | 5 | 0 | 0 | 5 | 0 | / |
| Control 14 | retarded | 53 | 0 | 0 | 35 | 18 | euploid |
| Control 15 | retarded | 35 | 0 | 0/1 | 27 | 7 | euploid (mosaic) |
| Putrescine 3 | retarded | 29 | 0 | 0 | 22 | 7 | euploid |
| Putrescine 4 | 1 | 28 | 0 | 1/2 | 17 | 9 | euploid (mosaic) |
| Putrescine 5 | retarded | 35 | 0 | 0 | 32 | 3 | euploid |
| Putrescine 6 | 2 | / | / | / | / | / | / |
| Putrescine 7 | 1 | 26 | 17/26 | 0 | 0 | 0 | triploid |
| Putrescine 8 | normal | 35 | 0 | 0 | 29 | 6 | euploid |
| Putrescine 9 | 2 | / | / | / | / | / | / |
| Putrescine 10 | 2 | / | / | / | / | / | / |
| Putrescine 11 | 1 | 34 | 11/34 | 0 | 0 | 0 | triploid |
| Control 16 | retarded | 43 | 0 | 1 | 35 | 7 | euploid (mosaic) |
| Control 17 | retarded | 35 | 0 | 1 | 29 | 5 | euploid (mosaic) |
| Control 18 | 1 | 16 | 0 | 0 | 9 | 7 | euploid |
|  |  |  | 40, 9×; 39, 5×; ≤38, 2× | | | | |
| Control 19 | retarded | 8 | 0 | 1 | 6 | 1 | / |
|  |  |  | 41 1×; 40 6×; 39 0×; ≤38 1× | | | | |
| Control 20 | retarded | 30 | 0 | 0 | 25 | 5 | euploid |
| Control 21 | retarded | 31 | 0 | 0 | 19 | 12 | euploid |
| Control 22 | 1 | 36 | 0 | 0 | 33 | 3 | euploid |
| Control 23 | 2 | 0 | 0 | 0 | 0 | 0 | / |
| Control 24 | 1 | 33 | 0 | 1 | 29 | 3 | euploid (mosaic) |
| Control 25 | retarded | 15 | 0 | 0 | 10 | 5 | euploid |
|  |  |  | 40, 10×; 39, 1×; ≤38, 4× | | | | |
| Control 26 | normal | 30 | 0 | 0 | 27 | 3 | euploid |
| Control 27 | normal | 30 | 0 | 0 | 27 | 3 | euploid |
| Control 28 | retarded | 28 | 0 | 0 | 27 | 1 | euploid |
| Control 29 | 2 | / | / | / | / | / | / |
| Control 30 | 2 | / | / | / | / | / | / |
| Control 31 | 2 | / | / | / | / | / | / |
| Control 32 | 2 | / | / | / | / | / | / |
| Control 33 | 2 | / | / | / | / | / | / |
| Control 34 | 1 | 32 | 0 | 1 | 27 | 4 | euploid (mosaic) |
| Control 35 | 1 | 30 | 0 | 1/2 | 16 | 12 | euploid (mosaic) |
|  |  |  | 42, 1×; 41, 1×; 40, 16×; 39, 5×; ≤38, 7× | | | | |
| Control 36 | 1 | 31 | 0 | 0 | 26 | 5 | euploid |
| Control 37 | 2 | / | / | / | / | / | / |
| Control 38 | 1 | 35 | 0 | 1 | 29 | 5 | euploid (mosaic) |
| Control 39 | normal | 30 | 0 | 0 | 19 | 11 | euploid |
| Control 40 | 2 | / | / | / | / | / | / |
| Control 41 | retarded | 30 | 0 | 0 | 22 | 8 | euploid |
| Control 42 | 1 | 34 | 0 | 0 | 29 | 5 | euploid |
| Control 43 | retarded | 33 | 0 | 14/17 | 8 | 8 | hyperploids (41) |
| Control 44 | retarded | 27 | 0 | 1 | 21 | 5 | euploid (mosaic) |
| Control 45 | 2 | 0 | 0 | 0 | 0 | 0 | / |
| Control 46 | 1 | 28 | 0 | 0 | 21 | 7 | euploid |
| Putrescine 12 | 1 | 36 | 0 | 1 | 26 | 9 | euploid (mosaic) |
| Putrescine 13 | normal | 30 | 0 | 2 | 20 | 8 | euploid (mosaic) |
| Putrescine 14 | 2 | / | / | / | / | / | / |
| Putrescine 15 | retarded | 34 | 0 | 1/2 | 27 | 5 | euploid (mosaic) |
| Putrescine 16 | retarded | 32 | 0 | 0 | 25 | 7 | euploid |
| Putrescine 17 | retarded | 33 | 0 | 0 | 25 | 8 | euploid |
| Putrescine 18 | retarded | 33 | 0 | 0/1 | 28 | 4 | euploid (mosaic) |
| Putrescine 19 | 1 | 44 | 27/43 | 0 | 0 | 1 | triploid |
| Putrescine 20 | 1 | 23 | 0 | 2 | 15 | 6 | euploid (mosaic) |
| Putrescine 21 | retarded | 51 | 0 | 0/1 | 41 | 9 | euploid (mosaic) |
| Putrescine 22 | 1 | 40 | 0 | 0 | 30 | 10 | euploid |
| Putrescine 23 | retarded | 34 | 0 | 1 | 28 | 5 | euploid (mosaic) |
| Putrescine 24 | retarded | 42 | 0 | 1 | 32 | 9 | euploid (mosaic) |
| Putrescine 25 | retarded | 49 | 0 | 1/4 | 38 | 7 | euploid (mosaic) |
| Control 47 | normal | 44 | 0 | 1 | 35 | 8 | euploid (mosaic) |
| Control 48 | normal | 54 | 0 | 0 | 48 | 6 | euploid |
| Control 49 | normal | 27 | 0 | 0 | 23 | 4 | euploid |
| Control 50 | normal | 46 | 0 | 1 | 36 | 9 | euploid (mosaic) |
| Control 51 | normal | 49 | 0 | 0 | 37 | 12 | euploid |
| Putrescine 26 | 2 | 0 | 0 | 0 | 0 | 0 | / |
| Putrescine 27 | 1 | 35 | 0 | 2 | 25 | 8 | euploid (mosaic) |
| Putrescine 28 | normal | 48 | 0 | 0 | 40 | 8 | euploid |
| Putrescine 29 | normal | 12 | 0 | 0 | 11 | 1 | euploid |
| Putrescine 30 | retarded | 51 | 0 | 33/34 | 12 | 5 | hyperploids (41) |
| Putrescine 31 | normal | 50 | 0 | 0 | 40 | 10 | euploid |
| Putrescine 32 | 2 | 0 | 0 | 0 | 0 | 0 | / |
| Putrescine 33 | normal | 25 | 0 | 0 | 19 | 6 | euploid |
| Putrescine 34 | normal | 50 | 0 | 0 | 39 | 11 | euploid |
| Putrescine 35 | retarded | 41 | 0 | 0 | 37 | 4 | euploid |
| Putrescine 36 | 2 | / | / | / | / | / | / |
| Putrescine 37 | retarded | 61 | 0 | 0 | 46 | 15 | euploid |
| Putrescine 38 | retarded | 12 | 0 | 8 | 3 | 1 | Hyperploids (41) |
| Putrescine 39 | retarded | 60 | 0 | 0 | 49 | 11 | euploid |
| Putrescine 40 | 1 | 49 | 0 | 2/42 | 3 | 4 | hyperploids (43) (30/42) |
| Putrescine 41 | 1 | 64 | 0 | 0 | 54 | 10 | euploid |
| Putrescine 42 | retarded | 69 | 0 | 1 | 54 | 14 | euploid (mosaic) |
| Control 52 | retarded | 47 | 0 | 0 | 37 | 10 | euploid |
| Control 53 | retarded | 62 | 0 | 0 | 43 | 19 | euploid |
| Control 54 | 1 | 60 | 0 | 1 | 50 | 9 | euploid (mosaic) |
| Control 55 | retarded | 32 | 0 | 0 | 24 | 8 | euploid |
| Control 56 | 1 | 65 | 0 | 1 | 57 | 7 | euploid (mosaic) |
| Control 57 | 1 | 50 | 0 | 1 | 28 | 21 | euploid (mosaic) |
| Control 58 | 2 | 0 | 0 | 0 | 0 | 0 | / |
| Control 59 | 2 | / | / | / | / | / | / |
| Control 60 | 2 | 0 | 0 | 0 | 0 | 0 | / |
| Putrescine 43 | 2 | / | / | / | / | / | / |
| Control 61 | 2 | / | / | / | / | / | / |
| Control 62 | 2 | / | / | / | / | / | / |
| Putrescine 44 | retarded | 16 | 11/1 | 0 | 0 | 0 | triploid |
| Putrescine 45 | normal | 24 | 0 | 0 | 21 | 3 | euploid |
| Putrescine 46 | normal | 18 | 0 | 0 | 17 | 1 | euploid |
| Putrescine 47 | normal | 25 | 0 | 1/2 | 18 | 5 | euploid (mosaic) |
| Putrescine 48 | normal | 40 | 0 | 0 | 31 | 9 | euploid |
| Putrescine 49 | normal | 17 | 0 | 0 | 15 | 2 | euploid |
| Putrescine 50 | 2 | / | / | / | / | / | / |

*In this column, “0” means the fetus/resorption was karyotyped but no countable metaphase spread was obtained while “/” means the fetus/resorption was not karyotyped.

**The numerator denotes the number of cells containing 60 chromosomes while the denominator 58-62 chromosomes.

*** The numerator denotes the number of cells containing 41 chromosomes while the denominator 41-43 chromosomes.

Of the five hyperploids, four were with 41 chromosomes and one with 43 chromosomes (30 out of 49 countable spreads).

More details are provided for the few embryos that had excessive number of hypoploid cells.

**S1 Table.** Details of karyotypes of retarded/resorbed fetuses in 10-month-old C57BL/6 mice
