# Supplementary material for: The Majority of Resorptions in Old Mice Are Euploid
Source: PLoS One. 2015 Dec 4;10(12):e0143360. doi: 10.1371/journal.pone.0143360 (PMC4670076; doi:10.1371/journal.pone.0143360)
Supplement: S2 Table — (DOCX) [file pone.0143360.s002.docx]

| **Fetal code** | **Fetal status** | **Countable cells*** | **Karyotype in #dyads** | | | | **Diagnosis** |
| --- | --- | --- | --- | --- | --- | --- | --- |
|  |  |  | 60** | 41*** | 40 | ≤39 |  |
| 1 | 2 | 2 | 0 | 0 | 0 | 2 | / |
| 2 | 1 | 1 | 0 | 0 | 0 | 1 | / |
| 3 | 2 | 0 | 0 | 0 | 0 | 0 | / |
| 4 | 1 | 0 | 0 | 0 | 0 | 0 | / |
| 5 | 2 | 0 | 0 | 0 | 0 | 0 | / |
| 6 | 2 | 0 | 0 | 0 | 0 | 0 | / |
| 7 | 1 | 10 | 0 | 0 | 4 | 6 | / |
|  |  |  | 41 0×; 40 4×; 39 3×; ≤38 3× | | | | |
| 8 | 2 | 0 | 0 | 0 | 0 | 0 | / |
| 9 | 2 | / | / | / | / | / | / |
| 10 | 2 | / | / | / | / | / | / |
| 11 | 2 | 1 | 0 | 0 | 0 | 1 | / |
| 12 | 2 | 0 | 0 | 0 | 0 | 0 | / |
| 13 | 2 | 0 | 0 | 0 | 0 | 0 | / |
| 14 | 2 | 3 | 0 | 1 | 0 | 2 | / |
| 15 | 1 | 3 | 0 | 0 | 2 | 1 | / |
| 16 | 2 | 3 | 0 | 0 | 0 | 3 | / |
| 17 | 2 | 3 | 0 | 0 | 2 | 1 | / |
| 18 | 2 | 3 | 0 | 0 | 2 | 1 | / |
| 19 | 2 | 0 | 0 | 0 | 0 | 0 | / |
| 20 | 1 | 12 | 0 | 0 | 5 | 7 | euploid |
|  |  |  | 40, 5×; 39, 1×; 38, 1×; 37, 1×; ≤35 4× | | | | |
| 21 | 1 | 4 | 0 | 0 | 3 | 1 | / |
| 22 | 1 | 28 | 0 | 0 | 20 | 8 | euploid |
| 23 | 2 | / | / | / | / | / | / |
| 24 | 2 | / | / | / | / | / | / |
| 25 | 2 | / | / | / | / | / | / |
| 26 | 2 | 0 | 0 | 0 | 0 | 0 | / |
| 27 | 2 | 0 | 0 | 0 | 0 | 0 | / |
| 28 | 2 | / | / | / | / | / | / |
| 29 | 2 | 0 | 0 | 0 | 0 | 0 | / |
| 30 | 1 | 13 | 0 | 0 | 8 | 5 | euploid |
|  |  |  | 40, 8×; 39, 3×; ≤38, 2× | | | | |
| 31 | 2 | 0 | 0 | 0 | 0 | 0 | / |
| 32 | 2 | / | / | / | / | / | / |
| 33 | 1 | 29 | 0 | 0 | 21 | 8 | euploid |
| 34 | 1 | 23 | 0 | 1 | 15 | 7 | euploid (mosaic) |
| 35 | 1 | 24 | 0 | 18/19 | 0 | 5 | hyperploid |
| 36 | 1 | 5 | 0 | 0 | 1 | 4 | / |
| 37 | 1 | 24 | 0 | 0 | 21 | 3 | euploid |
| 38 | 1 | 24 | 0 | 0 | 18 | 6 | euploid |
| 39 | 1 | 30 | 0 | 0 | 26 | 4 | euploid |
| 40 | 2 | 0 | 0 | 0 | 0 | 0 | / |
| 41 | 1 | 25 | 0 | 0 | 15 | 10 | euploid |
| 42 | 2 | / | / | / | / | / | / |
| 43 | Retarded | 31 | 0 | 1 | 28 | 2 | euploid (mosaic) |
| 44 | 1 | 28 | 0 | 1/2 | 23 | 3 | euploid (mosaic) |
| 45 | Retarded | 30 | 0 | 0/1 | 19 | 10 | euploid (mosaic) |
| 46 | 1 | 8 | 0/3 | 0/1 | 0 | 4 | / |
| 47 | 1 | 28 | 0 | 0 | 26 | 2 | euploid |
| 48 | 2 | / | / | / | / | / | / |
| 49 | 2 | 0 | 0 | 0 | 0 | 0 | / |
| 50 | 2 | / | / | / | / | / | / |
| 51 | 2 | 0 | / | / | / | / | / |
| 52 | 1 | 0 | / | / | / | / | / |
| 53 | 1 | 38 | 0 | 0 | 30 | 8 | euploid |
| 54 | 1 | 44 | 0 | 0 | 32 | 12 | euploid |
| 55 | 1 | 44 | 0 | 1/2 | 27 | 15 | euploid (mosaic) |
| 56 | 1 | 42 | 0 | 1 | 34 | 7 | euploid (mosaic) |
| 57 | 1 | 24 | 0 | 0 | 11 | 13 | euploid |
|  |  |  | 40, 11×; 39, 4×; ≤38 9× | | | | |
| 58 | Retarded | 0 | 0 | 0 | 0 | 0 | / |
| 59 | 1 | 28 | 0 | 1 | 21 | 6 | euploid (mosaic) |
| 60 | 1 | 37 | 0 | 2 | 18 | 17 | euploid (mosaic) |
|  |  |  | 41, 2×; 40, 18×; 39, 9×; ≤38, 8× | | | | |
| 61 | 1 | 8 | 0 | 1 | 4 | 3 | / |
| 62 | 2 | / | / | / | / | / | / |
| 63 | 2 | / | / | / | / | / | / |
| 64 | 1 | 30 | 19/30 | 0 | 0 | 0 | triploid |
| 65 | 1 | 38 | 0 | 1/2 | 31 | 5 | euploid (mosaic) |
| 66 | 1 | 30 | 0/1 | 2 | 21 | 6 | euploid (mosaic) |
| 67 | 1 | 41 | 0 | 1/2 | 36 | 3 | euploid (mosaic) |
| 68 | 2 | 0 | 0 | 0 | 0 | 0 | / |
| 69 | 1 | 38 | 0 | 0 | 33 | 5 | euploid |
| 70 | 1 | 40 | 0 | 0 | 34 | 6 | eulpoid |
| 71 | 2 | 0 | / | / | / | / | / |
| 72 | 1 | 48 | 0 | 1 | 25 | 22 | euploid (mosaic) |
|  |  |  | 41, 1×; 40, 25×; 39, 8×; ≤38, 14× | | | | |
| 73 | 1 | 31 | 0 | 1/2 | 22 | 7 | euploid (mosaic) |
| 74 | 1 | 25 | 0 | 0/1 | 15 | 9 | euploid (mosaic) |
|  |  |  | 42, 1×; 40, 15×; 39, 2×; ≤38 7× | | | | |
| 75 | 1 | 0 | 0 | 0 | 0 | 0 | / |
| 76 | 1 | 25 | 0/5 | 0 | 14 | 6 | euploid |
| 77 | 1 | 40 | 0 | 0 | 18 | 22 | euploid |
|  |  |  | 40, 18×; 39, 6×; ≤38 16× | | | | |
| 78 | 1 | 39 | 0 | 0 | 26 | 13 | euploid |
| 79 | 1 | 7 | 0 | 0 | 2 | 5 | / |
| 80 | 1 | 46 | 0 | 2 | 31 | 13 | euploid (mosaic) |
| 81 | 1 | 52 | 0 | 1/2 | 36 | 14 | euploid (mosaic) |
| 82 | 1 | 47 | 0 | 0 | 40 | 7 | euploid |
| 83 | 2 | 0 | 0 | 0 | 0 | 0 | / |
| 84 | Retarded | 55 | 0 | 1/3 | 35 | 17 | euploid (mosaic) |
| 85 | Retarded | 50 | 0 | 1/2 | 38 | 10 | euploid (mosaic) |
| 86 | Retarded | 38 | 0 | 0 | 29 | 9 | euploid |
| 87 | 1 | 49 | 0 | 2/3 | 26 | 20 | euploid (mosaic) |

*In this column, “0” means the fetus/resorption was karyotyped but no countable metaphase spread was obtained while “/” means the fetus/resorption was not karyotyped.

**The numerator denotes the number of cells containing 60 chromosomes while the denominator 58-62 chromosomes.

*** The numerator denotes the number of cells containing 41 chromosomes while the denominator 41-44 chromosomes.

More details are provided for the few embryos that had excessive number of hypoploid cells.

**S2 Table.** Details of karyotypes of retarded/resorbed fetuses in 17-month-old C57BL/6 mice.
